# Limb lengthening in achondroplasia: a cost-effectiveness analysis using a Markov model

**DOI:** 10.1186/s13023-026-04397-0

**Published:** 2026-05-27

**Authors:** Pablo Oscar Roza Miguel, Antonio Leiva Gea, María Rabanal Rubio, Nuria García-Rodríguez, Eduardo González Fidalgo

**Affiliations:** 1 MBA Institute Chair of Medical and Biomechanical Research, University of Oviedo, Gijón, Spain; 2 MBA Institute, Gijón, Spain; 3 Achondroplasia Unit, Department of Orthopaedic Surgery and Traumatology, Hospital Universitario Virgen de la Victoria, Málaga, Spain; 4 Orthopaedic Surgery and Traumatology, Instituto de Investigación Biomédica de Málaga, Málaga, Spain; 5 Marketing and Market Research, University of Oviedo, Oviedo, Spain; 6 Business Administration, University of Oviedo, Oviedo, Spain

**Keywords:** Achondroplasia, Dwarfism, Body height, External fixator, Bone lengthening, Ilizarov, Economics, Cost, Cost-effectiveness, Quality of life

## Abstract

**Background:**

Disproportionate short stature is one of the most characteristic clinical traits of achondroplasia, a condition with a significant impact on patients’ autonomy that results in a series of limitations leading to poorer health-related quality of life. Limb lengthening surgery is an effective therapeutic alternative for increasing the stature of persons with achondroplasia. The purpose of this study was to evaluate the cost-effectiveness of bone lengthening in patients with achondroplasia and to estimate the economic impact of the procedure on the Spanish health system by means of a Markov model.

**Results:**

Patients with achondroplasia who undergo a limb lengthening procedure gain, on average, 5.954 quality-adjusted life years (QALYs) more than unoperated patients (27.197 vs. 21.243 QALYs). As the procedure entails a mean incremental cost of €49,480, the resulting incremental cost-effectiveness ratio (ICER) is €8,310/QALY. The probabilistic sensitivity analysis (PSA) performed yielded a mean quality of life gain of 6.287 QALYs and a mean cost increase of €49,131, which translates into an ICER of €7,814/QALY. The budget impact analysis was based on the hypothesis that all patients with achondroplasia born every year in the country used as a reference (Spain, *n* = 15) undergo the procedure. Under this assumption, a cumulative cost increase of €8,896,196 can be estimated over a 15-year period (€8,836,931 following the PSA).

**Conclusions:**

Limb lengthening surgery in patients with achondroplasia can be considered a cost-effective intervention according to the most commonly accepted willingness-to-pay thresholds in Spain and other Western countries.

## Background

Achondroplasia is the most common form of skeletal dysplasia among humans [1, 2]. With a prevalence between 3.7 and 4.6 cases per 100,000 births [3, 4], it is estimated to affect between 250,000 and 360,000 persons worldwide [1, 4, 5]. Those who live with the condition present with a mutation of the fibroblast growth factor receptor 3 gene (FGFR3) [4, 6, 7] that leads to an inhibitory gain of function in growth plate chondrocytes resulting in a decreased proliferation of chondroblasts in growth cartilages. This interferes with longitudinal bone growth and triggers a series of musculoskeletal alterations [5, 8].

Such alterations comprise disproportionate short stature with rhizomelia, macrocephaly, midface retrusion, lumbar hyperlordosis, short-fingered trident hands, genu varum, joint hyperlaxity and hypotonia [5]. They are also associated with various medical complications, which may be neurological (foramen magnum stenosis or hydrocephaly), respiratory (obstructive sleep apnea), orthopedic (hyperlordosis, stenosis, genu varum or chronic pain), otolaryngological (recurrent otitis or hearing loss), dental (maxillary hypoplasia) or cardiovascular, among others, in nature [5]. All of these complications could shorten these patients’ life expectancy by around 10 years as compared with the general population [9].

However distinctive these alterations may be, the most characteristic trait of achondroplasia is short stature, with mean adult height standing at 130 cm in males and 125 cm in females, with some variation between studies [5, 10–12]. Abnormally short stature limits the person’s autonomy for performing everyday activities such as self-care, moving around, using transport or driving, and usually requires adaptations in educational, domestic and occupational environments [1, 5, 13–17]. These limitations could result in psychosocial problems, low self-esteem, isolation, difficulties in engaging in social interaction, and even depression [5, 17, 18], which may result in lower scores in questionnaires rating respondents’ health-related quality of life [19–23].

Despite the significant advances made in the last few years in the pharmacological treatment of achondroplasia, including the development of FGFR3-targeted therapies such as vosoritide [24–29], limb lengthening surgery remains an effective treatment option to increase the stature of these patients [30], it is also the only option capable of increasing the subjects’ stature to levels that may significantly improve their quality of life [22, 31].

**Fig. 1:**
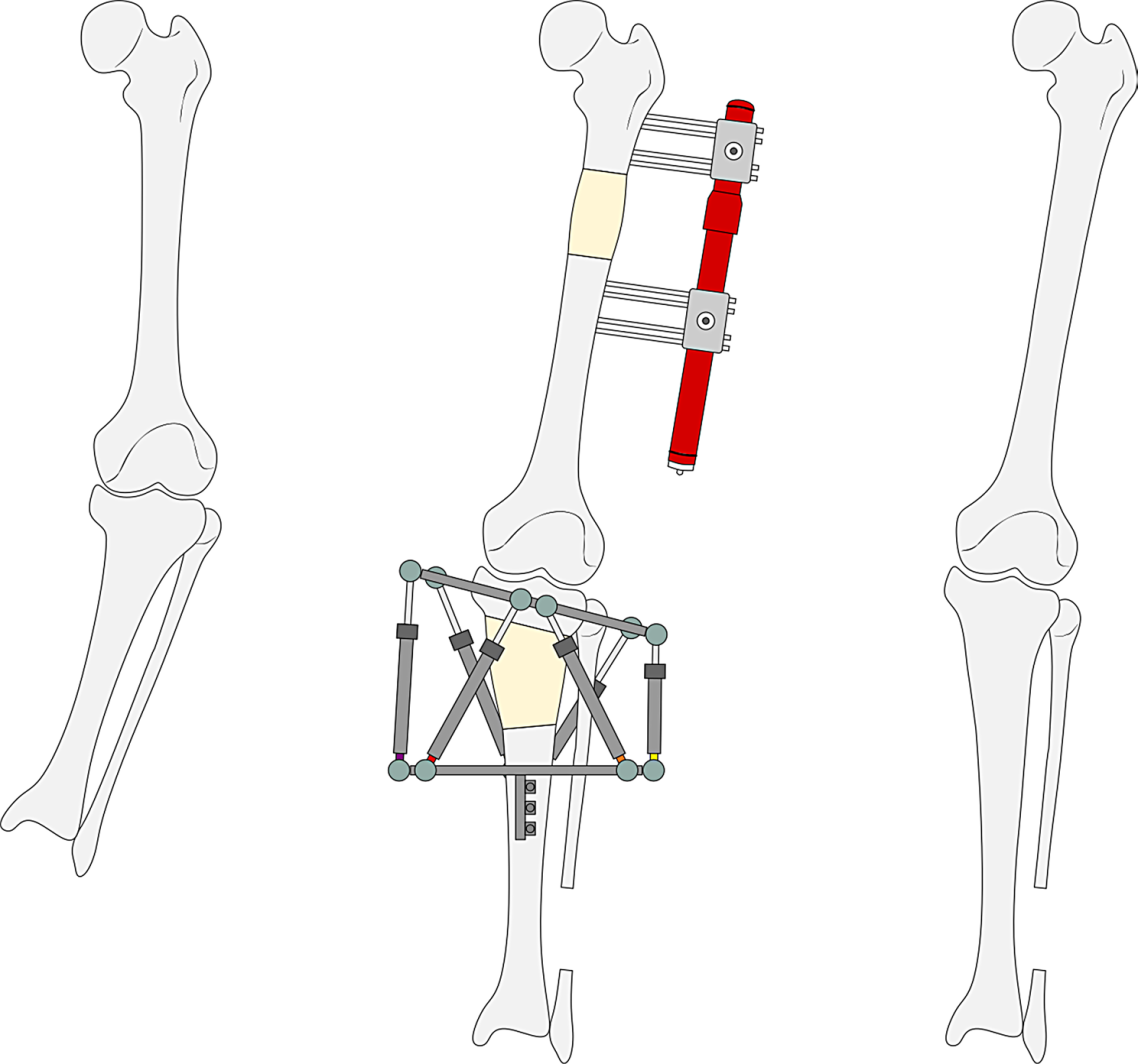
Femoral and tibial lengthening procedure with a monolateral fixator on the femur (Monotube Triax, Stryker Corp, United States) and a hexapod circular frame on the tibia (TrueLok Hex, Orthofix Srl, Italy)

Limb lengthening is carried out following the technique known as distraction osteogenesis or callotasis [32–36], which makes it possible to generate new bone between the two sides of an osteotomy, which are gradually drawn apart. The procedure can be performed using either internal or external fixation devices, but in the case of achondroplasia it is customary to use external fixators as they allow simultaneous correction of angular and rotational deformities in the limbs (Fig. 1) [14].

No economic evaluation studies have been published to date which demonstrate whether bone lengthening can be considered a cost-effective alternative for patients with achondroplasia. In view of the technique’s potential repercussions both on healthcare resources and on patients’ quality of life, the purpose of this study is to provide a comprehensive economic analysis of the cost-effectiveness of bone lengthening in persons with achondroplasia, as well as a budget impact analysis of the use of this treatment in patients suffering from the condition.

## Materials and methods

### Target population, perspective and time horizon

The target population of this economic evaluation comprised pre-adolescent individuals diagnosed with achondroplasia who were deemed eligible for limb lengthening surgery.

The analysis was conducted from the perspective of the Spanish Health System, considering the direct healthcare costs associated with the procedure, the clinical follow-up and any potential complications.

To capture the long-term economic and quality-of-life impact of the strategies under comparison, a time horizon was selected that encompassed the entire life expectancy of the individuals included in the model.

### Comparators

A comparison was made between two strategies used to address the short stature of individuals with achondroplasia:

- [No treatment]: Subjects satisfied with their stature or those who, despite being unsatisfied, prefer not to undergo a surgical procedure.
- [Surgical limb lengthening]: Individuals who decide to undergo a two-stage bone lengthening procedure [14]. The first stage comprises simultaneous lengthening of both femora and tibias. The second one consists in bilateral humeral lengthening.

### Markov model

A Markov model is a stochastic system that describes the evolution of a disease through a finite number of mutually exclusive health states, with transitions between them over the course of previously-defined time cycles. These constructs are particularly useful to model chronic or long-standing conditions characterized by repetitive or irreversible events [37].

**Fig. 2:**
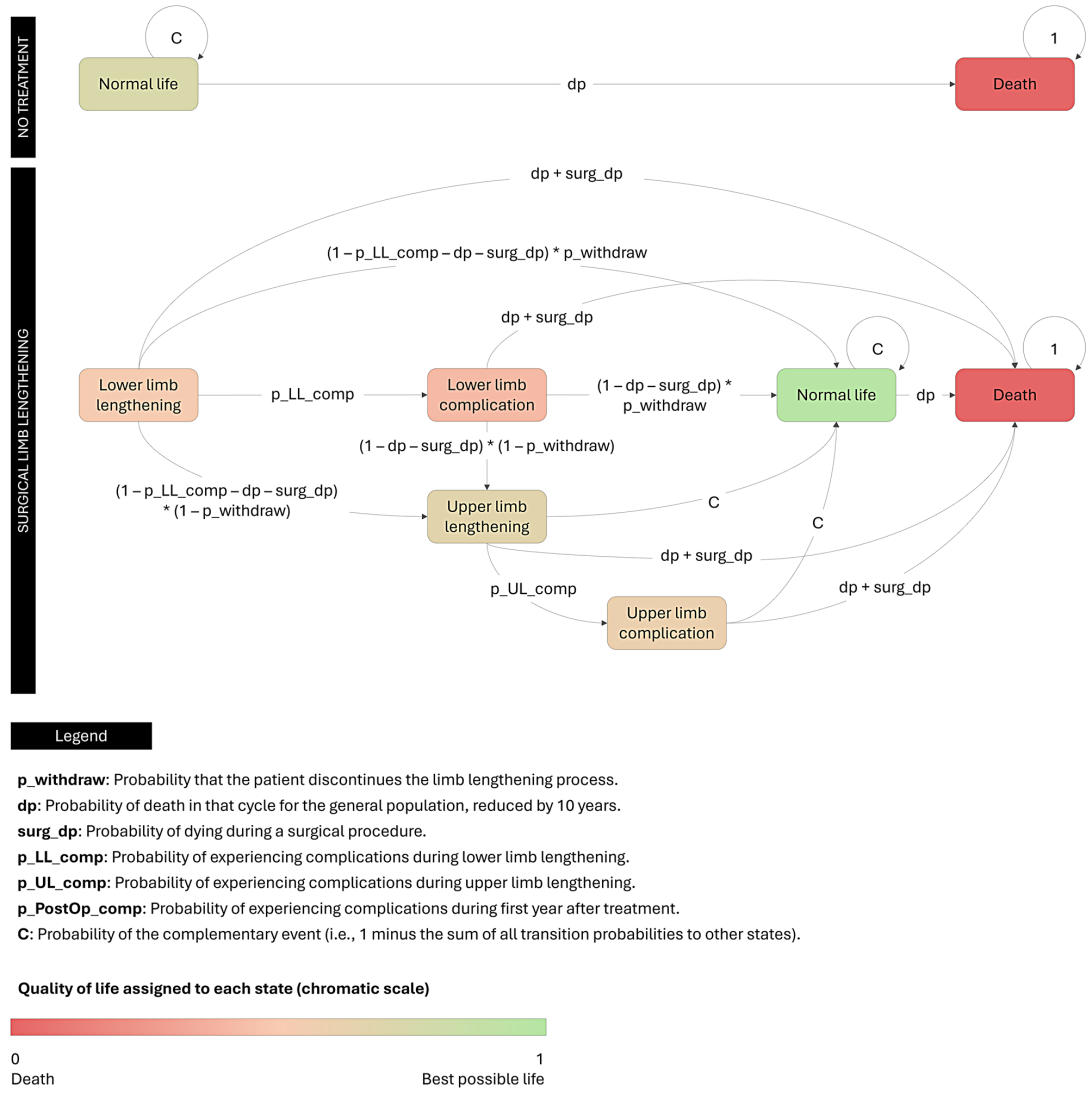
State-transition diagram of the proposed model

A Markov model was designed to compare the two different strategies (Fig. 2). Patients transition between seven different states through year-long cycles over a 90-cycle horizon. The transition matrices for each strategy are summarized in Tables 1 and 2. The model was designed and analyzed using the *heemod* and *BCEA* packages within the R statistical software package [38, 39].

States under the [No treatment] strategy:

- {Normal life}: Patients live in this state until their death. Their baseline quality of life is maintained over time, with adjustments according to a given discount rate. No cost differences were considered with respect to the limb lengthening strategy.
- {Death}: Absorbing state representing the patient’s death.

**Table 1:** No treatment strategy transition matrix

From / To	Normal life	Death
Normal life	1 - dp	dp
Death	0	1

* dp: Probability of death in that cycle for the general population, reduced by 10 years

States under the [Surgical limb lengthening] strategy:

- {Lower limb lengthening}: Simultaneous bilateral lengthening of both femora and tibias. This results in a temporary quality of life reduction and certain costs associated with the procedure and clinical follow-up. If the patient experiences complications, they transition to {Lower limb complication}. In the absence of complications, they may continue with their treatment toward {Upper limb lengthening} or discontinue it and move on to {Normal Life}. From this state, the patient may transition to {Death} according to their probabilities of a natural death compounded by those of dying during surgery [40].
- {Lower limb complication}: Complications resulting from lower limb lengthening, with their associated disutility and costs [41]. From this state, the patient may choose to carry on with their treatment {Upper limb lengthening} or to discontinue it {Normal life}. Or they may die {Death}.
- {Upper limb lengthening}: Bilateral humeral lengthening. It entails disutilities and costs derived from the procedure and clinical follow-up. If the patient experiences complications, they will transition to {Upper limb complications}. Otherwise, they will move on to {Normal life}. {Death} is also a possibility.
- {Upper limb complications}: Treatment of the potential complications following humerus lengthening, with their associated costs and disutilities. If the patient does not die {Death}, they will transition to {Normal life}.
- {Normal life}: According to the study used as a basis for this analysis, the patients’ quality of life exhibits an improvement with respect to the baseline level [42], with yearly adjustments according to a given discount rate. No cost differences were considered with respect to the nonsurgical strategy. The patient remains in this state until their death {Death}.
- {Death}: Absorbing state representing the patient’s death.

**Table 2:** Limb lengthening strategy transition matrix

From / To	Lower limb lengthening	Lower limb complications	Upper limb lengthening	Upper limb complications	Normal life	Death
Lower limb lengthening	0	p_LL_comp	(1 – p_LL_comp – dp – surg_dp) * (1 – p_withdraw)	0	(1 – p_LL_comp – dp – surg_dp) * p_withdraw	dp + surg_dp
Lower limb complications	0	0	(1 – dp – surg_dp) * (1 – p_withdraw)	0	(1 – dp – surg_dp) * p_withdraw	dp + surg_dp
Upper limb lengthening	0	0	0	p_UL_comp	1 – p_UL_comp – dp – surg_dp	dp + surg_dp
Upper limb complications	0	0	0	0	1 – dp – surg_dp	dp + surg_dp
Normal life	0	0	0	0	1 – dp	dp
Death	0	0	0	0	0	1

* dp: Probability of death in that cycle for the general population, reduced by 10 years

* surg_dp: Probability of dying during the surgical procedure

* p_LL_comp: Probability of experiencing complications during lower limb lengthening

* p_UL_comp: Probability of experiencing complications during upper limb lengthening

* p_withdraw: Probability that the patient discontinues the limb lengthening procedure

### Sources of information

The sources of information for the model are summarized in Table 3.

**Table 3:** Sources of information

Parameter	Base-case value	Distribution (PSA)	Source
QoL: Non-lengthened individuals	0.711 ± 0.266	Beta	Leiva (2022) [42]
QoL: Individuals who undergo LLL	0.888 ± 0.111	Beta	Leiva (2022) [42]
QoL: Individuals who undergo lower and ULL	0.944 ± 0.078	Beta	Leiva (2022) [42]
Life expectancy - General population	-	-	INE [43]
Life expectancy - Achondroplasia	-10 years	-	Wynn (2007) [9]
Elective pediatric orthopedic surgery mortality rate	0.013%	-	Chang (2012) [40]
Probability of reoperation: LLL	0.8	Beta	Leiva (2022) [42]
Probability of reoperation: ULL	0.1	Beta	Leiva (2022) [42]
Probability of LLL only	0.1	Beta	Expert opinion (AD-VVUH)
Utility multiplier during LLL surgery	0.7	Beta	Expert opinion (AD-VVUH)
Utility multiplier during ULL surgery	0.8	Beta	Expert opinion (AD-VVUH)
Utility multiplier during LLL complication surgery	0.8	Beta	Expert opinion (AD-VVUH)
Utility multiplier during ULL complication surgery	0.75	Beta	Expert opinion (AD-VVUH)
Mean cost: LLL procedure	28,539 €	Gamma	Price lists (SAS), AD-VVUH data, and public tender data [44, 45]
Mean cost: ULL procedure	10,834 €	Gamma	Price lists (SAS), AD-VVUH data, and public tender data [44, 45]
Mean cost: Complication procedure	6,275 €	Gamma	Price lists (SAS), AD-VVUH data, and public tender data [44, 45]
Discount rate on costs	3%	-	Spanish Ministry of Health (2023) [46]
Discount rate on health outcomes	3%	-	Spanish Ministry of Health (2023) [46]
Willingness-to-pay threshold	25,000 €	-	Sacristan (2020) [47]
Number of new cases per year	up to 15	-	Foreman (2020) applied to INE data [4, 43]

LLL: Lower limb lengthening

ULL: Upper limb lengthening

AD-VVUH: Achondroplasia Unit, Virgen de la Victoria University Hospital (Málaga, Spain)

SAS: Servicio Andaluz de Salud (Andalusian Health Service)

INE: Spanish National Institute of Statistics

The health outcomes of achondroplastic patients, as measured by means of the EQ-5D-Y [48], were obtained from Leiva et al. [42], who compared the quality of life of subjects with achondroplasia subjected to limb lengthening surgery with that of untreated individuals. In view of the absence of published data on the disutilities resulting from limb lengthening surgery and its complications, such disutilities were modeled based on the expert opinion of the medical team of the Achondroplasia Department of the Virgen de la Victoria University Hospital (AD-VVUH). The feedback from the largest patient association in Spain (Alpe Foundation) was also collected. In the absence of long-term follow-up data, the incremental quality-of-life gain associated with limb lengthening compared with no treatment was assumed to persist over the patients’ lifetime, with outcomes discounted according to standard rates.

An analysis was made of the direct costs of the two strategies under comparison, including those related to preoperative visits, imaging studies, operating room use, medical team, implants, hospital stay, follow-up visits and pin site care. The cost of each healthcare resource was obtained from the Andalusian Health Service’s Public Price List [44], and the cost of implants was taken from those quoted in a recent public tender [45]. The number of resources used under each of the headings above was calculated based on the Achondroplasia Unit of the Virgen de la Victoria University Hospital’s experience.

The likelihood of complications and their severity were taken from the clinical study that served as a basis for the present analysis [42]. The probability of dying by age was taken from the mortality tables published by the Spanish National Institute of Statistics (Instituto Nacional de Estadística, INE) [43] reduced by 10 years [9], while the probability of dying during an elective pediatric orthopedic surgical procedure was extracted from Chang et al. [40]. The probability of discontinuation of the limb lengthening process was determined based on the AD-VVUH’s experience.

The discount rate employed in the study, with respect to costs and health outcomes, was 3% in both cases [46].

### Decision criterion

The economic evaluation of the two strategies was carried out using the incremental cost-effectiveness ratio (ICER), comparing the value yielded by the model with the threshold defined for Spain by Sacristan et al. [47] and with the threshold defined by NICE, considered the main international reference in this regard [49].

### Budget impact analysis

The budget impact analysis considered a 15-year time horizon. Based on recent birth rate statistics from Spain [43] and on the reported incidence of achondroplasia [3, 4], it was estimated that around 15 new persons with the condition are likely to join the system every year. Although most probably not all of them end up undergoing limb lengthening, the assumption was made that all patients would undergo the procedure as it was thought best to be conservative and consider the maximum potential expense for the Spanish Heath System.

### Sensitivity analysis

A probabilistic sensitivity analysis (PSA) was conducted for the basic (deterministic) Markov model with a view to incorporating the uncertainty associated with utilities, costs and transition probabilities. Utilities were modeled through a beta distribution, parameterized by the available mean and standard deviation values. Healthcare costs were modeled through a gamma distribution, parameterized by mean and standard deviation. In the absence of parameter-specific variance estimates, a coefficient of variation of 30% was assumed for all cost parameters to reflect uncertainty. Transition probabilities were also modeled through a beta distribution. A fixed effective sample size (*n* = 50) was used to reflect moderate uncertainty. The number of Monte Carlo iterations in the PSA stood at 1,000.

## Results

**Fig. 3:**
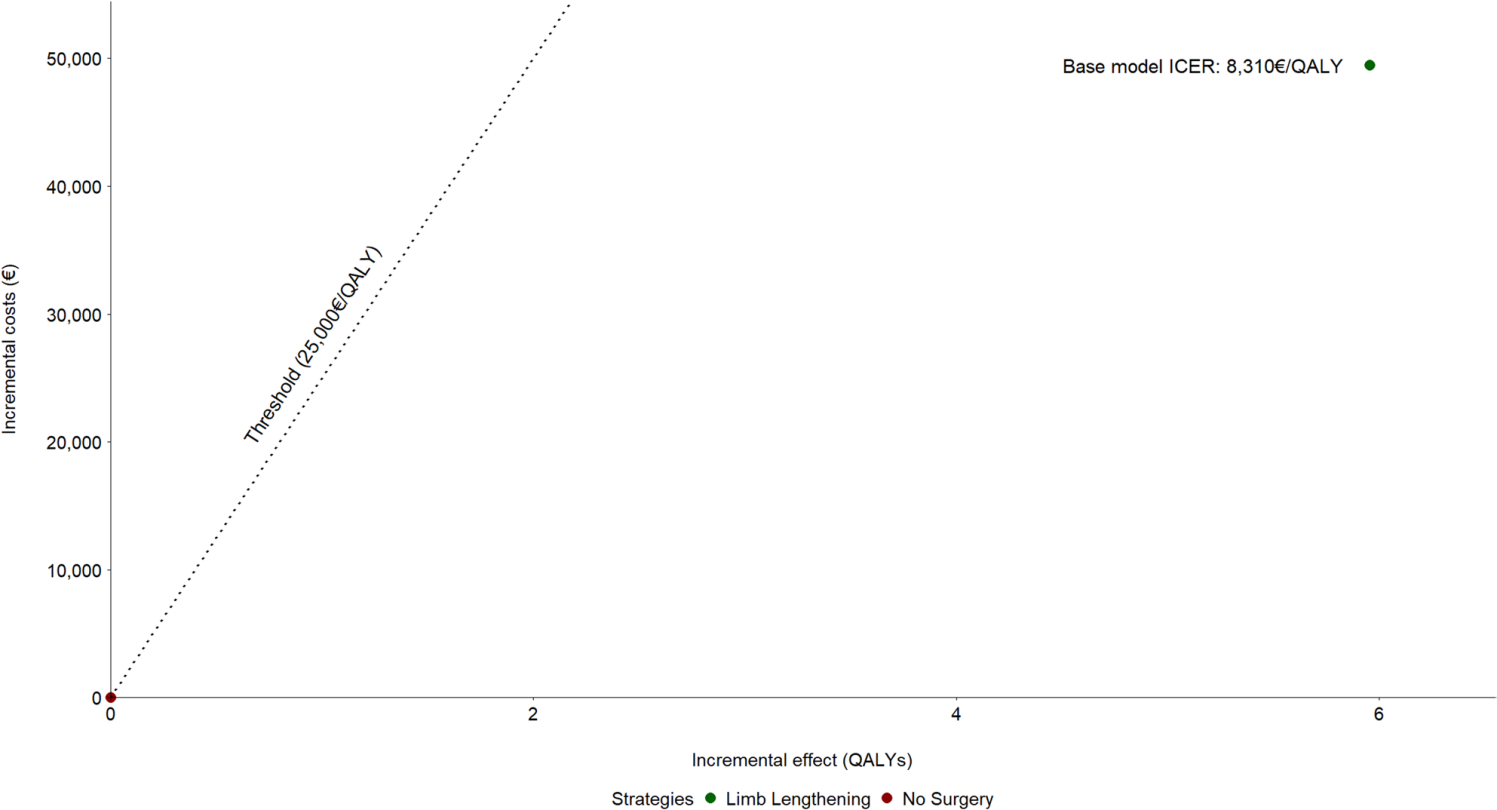
Cost-effectiveness plane for the baseline model showing that the limb lengthening strategy is cost-effective at a willingness-to-pay threshold of €25,000/QALY

According to the basic Markov model, a patient with achondroplasia who chooses not to undergo limb lengthening accumulates, on average, 21.243 quality-adjusted life years (QALYs). In turn, those who do undergo the treatment accumulate 27.197 QALYs over their lifetime, representing a gain of 5.954 QALYs attributable to the procedure. As regards the mean total costs, those associated with limb lengthening are €49,480 per patient and those associated with the non-surgical strategy are considered null as they coincide with some of the costs corresponding to the operative strategy. This results in an ICER of €8,310/QALY, indicating that the limb lengthening strategy is cost-effective at the willingness-to-pay (WTP) threshold selected for the study (Fig. 3).

**Fig. 4:**
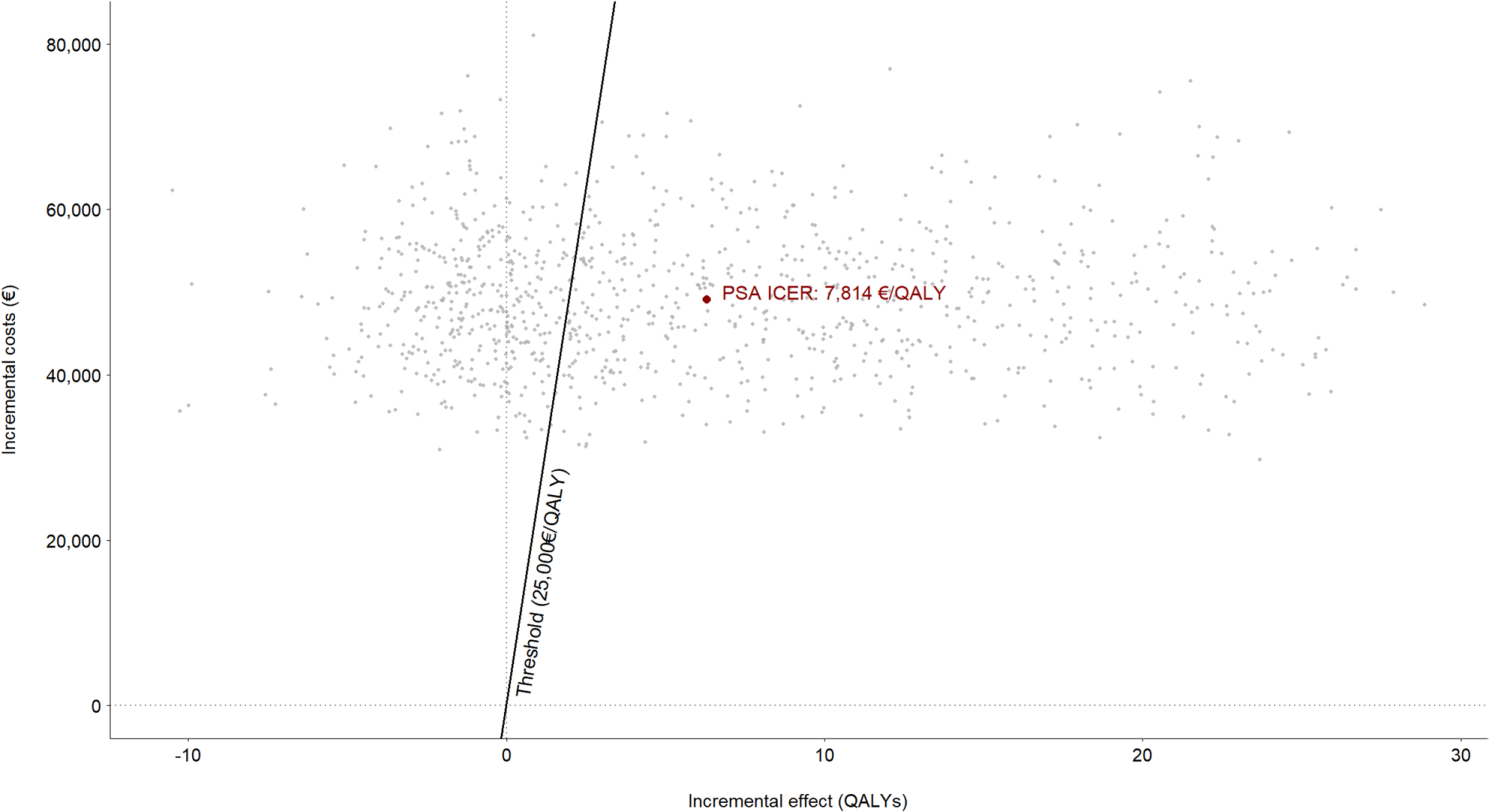
Cost-effectiveness plane for the probabilistic sensitivity model, showing that limb lengthening is cost-effective at an acceptability threshold of €25,000/QALY

According to the PSA, patients not undergoing limb lengthening surgery benefit, on average, from a cumulative utility of 20.898 QALYs, with no additional associated economic costs. In turn, those who do undergo surgical treatment accumulate, on average, a total of 27.185 QALYs throughout their lifetime, at a mean total incremental cost of €49,131 per patient. This means that the difference between the two strategies is estimated at 6.287 QALYs, which translates into an ICER of €7,814/QALY. These results confirm that, according to the findings of the PSA, limb lengthening is a cost-effective strategy (Fig. 4).

**Fig. 5:**
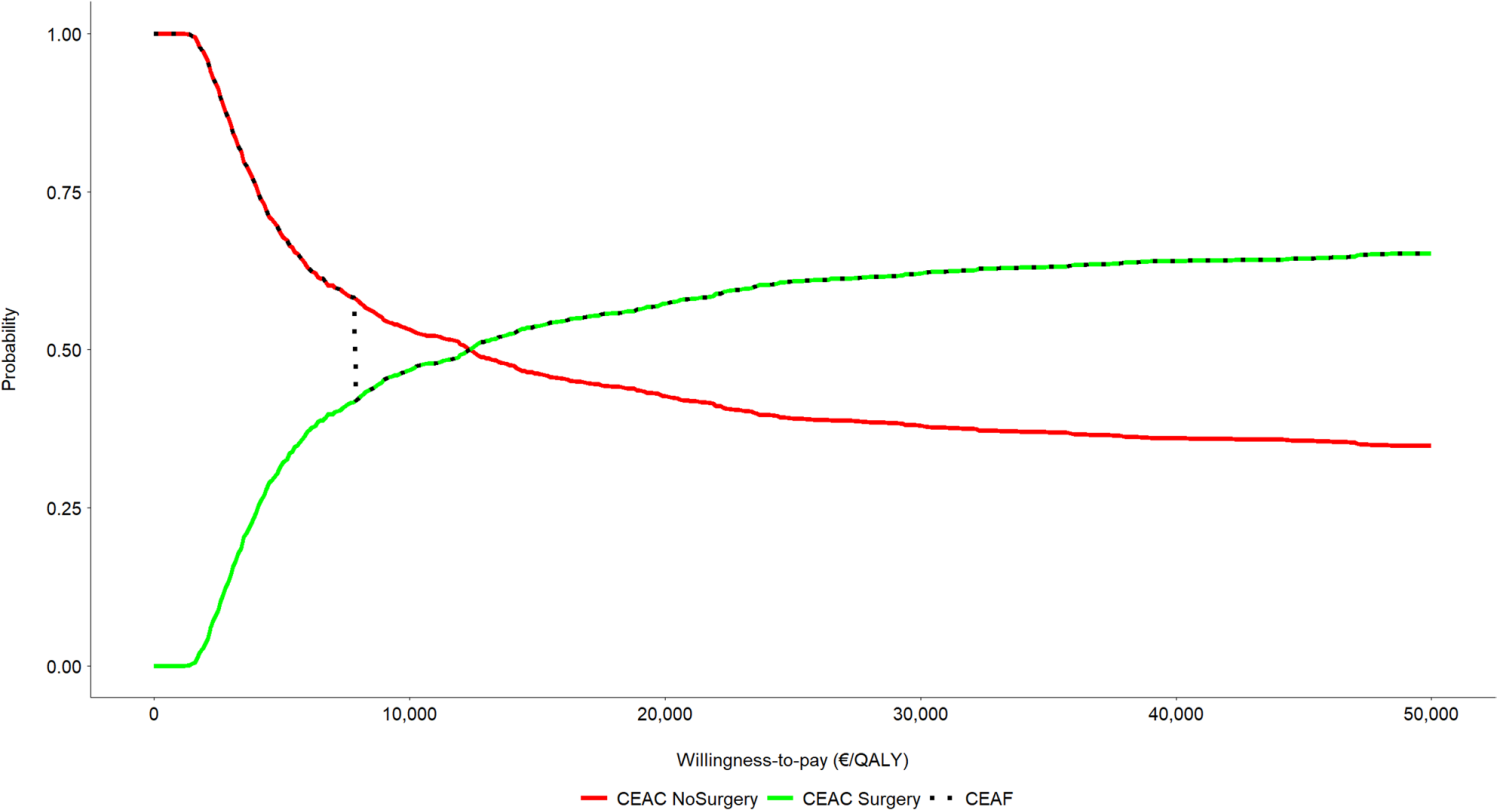
Cost-effectiveness acceptability curves (CEACs) of the two strategies under comparison are illustrated together with the cost-effectiveness acceptability frontier (CEAF). Limb lengthening surgery has a higher probability of being cost-effective than the nonsurgical option from approximately €13,000/QALY upwards, and it is considered the better strategy in terms of expected monetary benefit from €7,814/QALY upwards

Figure 5 illustrates the cost-effectiveness acceptability curve (CEAC) and the cost-effectiveness acceptability frontier (CEAF) derived from the PSA. At low WTP thresholds, the nonsurgical strategy is more likely to yield a higher net monetary benefit. As the WTP threshold increases, so does the probability that the limb lengthening strategy may be the one offering a higher net monetary benefit, exceeding that of not undergoing surgery by around €13,000/QALY. Given the uncertainty and the variability regarding costs and effects, the WTP threshold above which a strategy becomes optimal in terms of expected value does not necessarily coincide with the threshold at which it is associated with a 50% probability of being cost-effective. For that reason, there is no exact overlap between the CEAF and the upper envelope of the two CEACs.

In fact, the net monetary benefit at a WTP threshold of €25,000 stands at €522,460 for an unoperated patient and at €630,506 for one subjected to limb lengthening. The fact that the expected net monetary benefit is higher for surgery reinforces the idea that the limb lengthening strategy is cost-effective at the specified threshold.

**Fig. 6:**
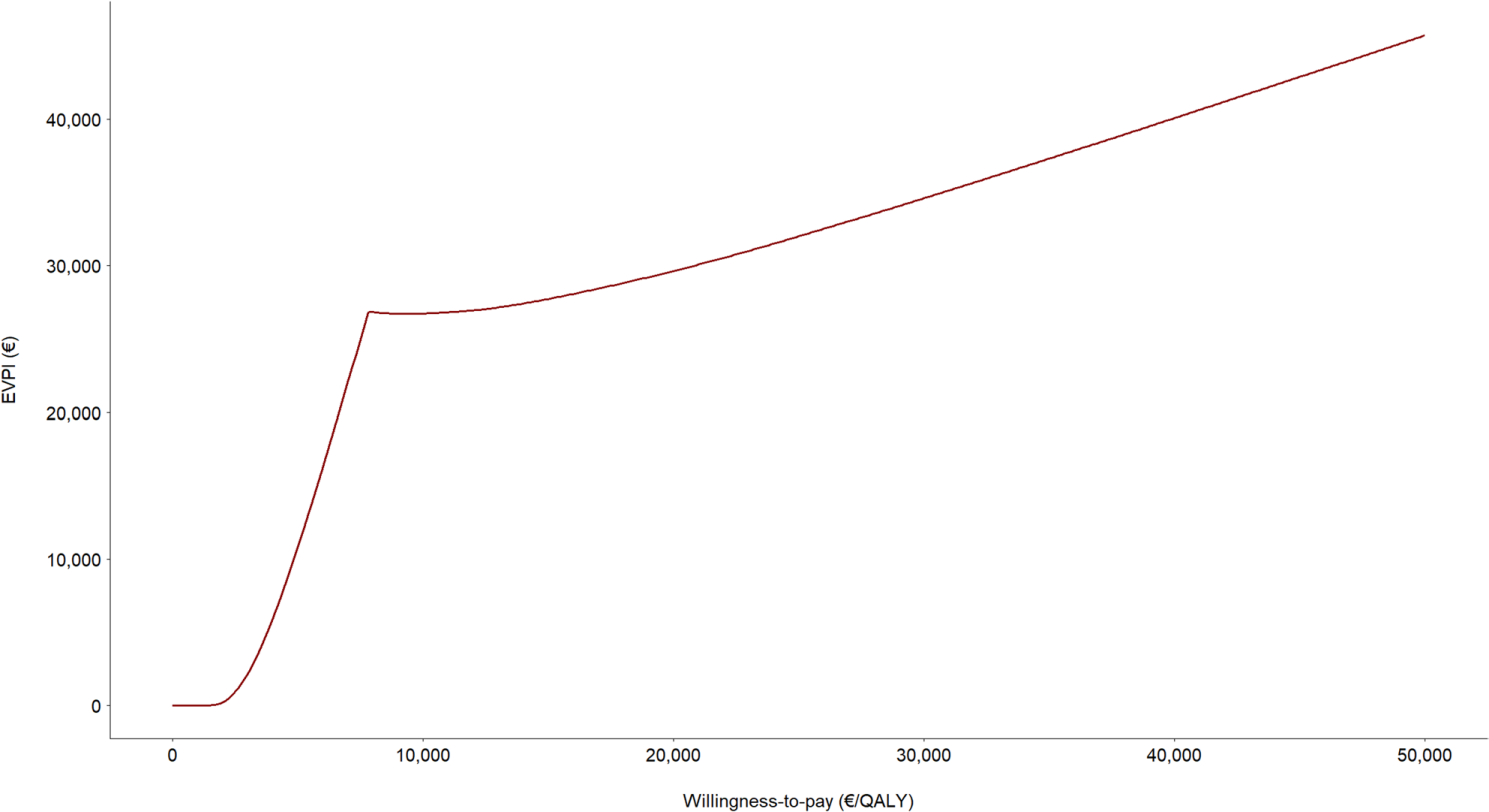
Expected value of perfect information (EVPI), showing a marked EVPI growth at low willingness-to-pay thresholds. The rising trend slows down once the EVPI reaches the ICER value corresponding to the limb lengthening strategy

Figure 6 shows the expected value of perfect information (EVPI) for different WTP thresholds. For low WTP thresholds –where the results of these analyses are practically irrelevant for payers– the EVPI is practically nil, as it is almost certain that the nonsurgical strategy will be chosen. Nonetheless, as the WTP threshold rises, the EVPI experiences a marked growth, reaching the highest values where the WTP threshold coincides with the ICER calculated by the model (€7,814/QALY), where the uncertainty regarding the decision is greatest. From that point onwards, most likely due to the uncertainty around quality-of-life outcomes, the EVPI increase slows down, suggesting that the conduction of additional studies intended to reduce payers’ uncertainty may be warranted.

**Fig. 7:**
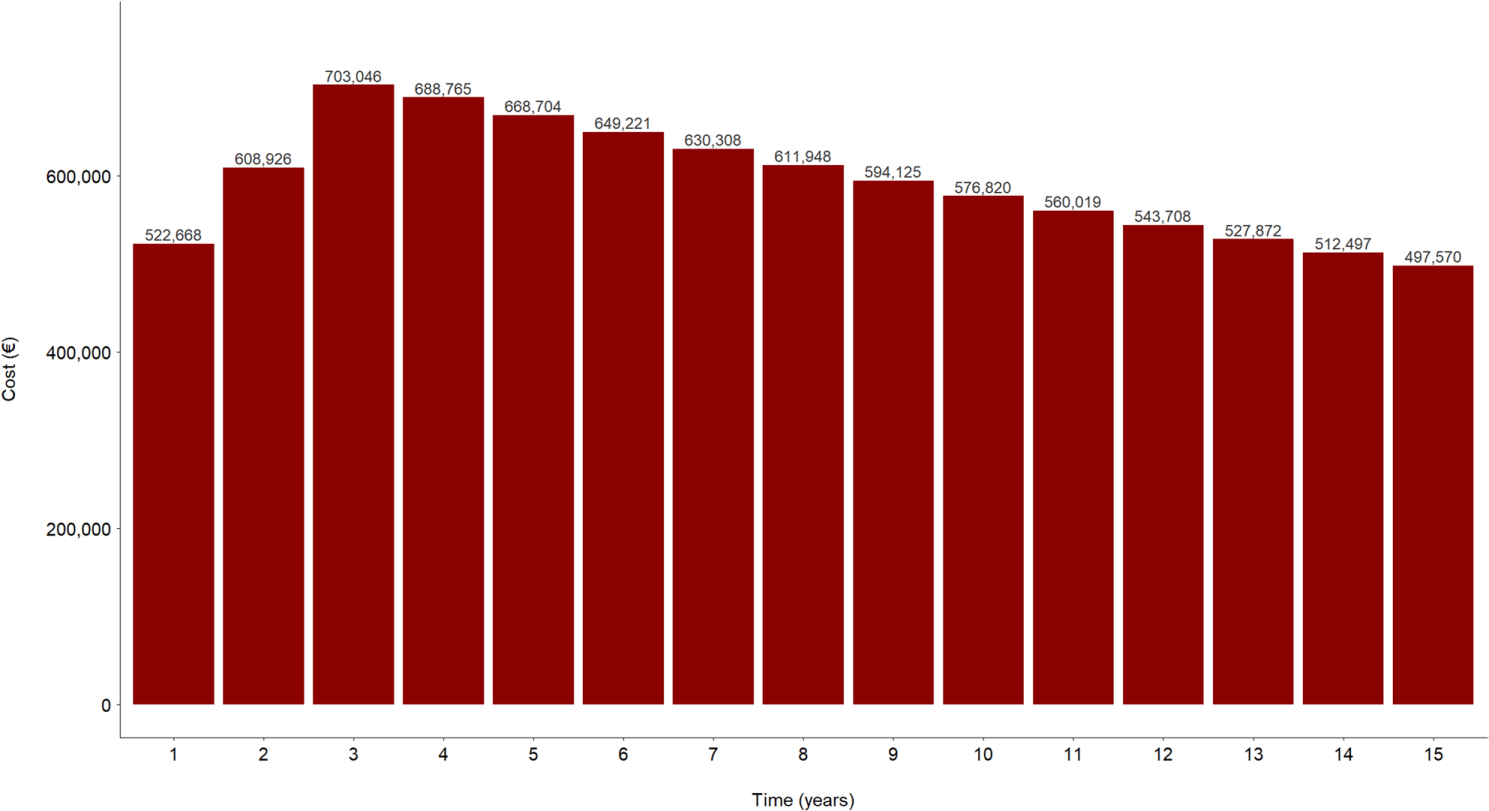
Incremental spending over a 15-year period following implementation of limb lengthening programs, under the assumption that all the patients with achondroplasia in the country participate in such programs

As regards the budget impact analysis (Fig. 7), under the worst-case scenario that 15 patients are treated every year, the model estimates a cumulative additional expenditure of €8,896,196 over the 15-year time horizon due to the implementation of the limb lengthening strategy in patients with achondroplasia. Following application of the PSA, this cost was estimated at €8,836,931. A scenario analysis assuming that only 50% of the eligible population (*n* = 7 patients per year) undergoes the procedure yielded a cumulative cost of €4,151,558 over the same 15-year period (€4,176,769 following the PSA), as expected under a proportional reduction in the treated population.

## Discussion

Limb lengthening surgery is a complex procedure that comes with certain risks but which, in patients with achondroplasia, is associated with various benefits [5, 50–54]. The operation’s most obvious result is an increase in stature and arm span, as well as an improvement in body proportions that often leads to greater social acceptance and/or increased self-esteem [5, 51, 55, 56]. But the cosmetic benefits of the procedures are by no means the only ones. The added height and the increased range of motion of the upper limbs provide patients with greater autonomy and improved function [1, 16, 17, 31, 57–59]. Moreover, the correction of the mechanical axes resulting from the procedure typically leads to improved gait and may reduce knee pain [1, 15, 52, 60]. Furthermore, the rhizomelic pattern associated with achondroplasia typically improves as a result of the operation [51]. All the above is likely to result in an improvement in health-related quality of life.

The multiplicity of potential benefits resulting from limb lengthening typically makes individuals prioritize some of them over the others, which means that the perception of the need to undergo this treatment typically varies across patients and their families as a result of external factors such as social pressure, standard medical practice or recommendations of patient associations [1, 5, 56]. In Spain, the official position of the Alpe Foundation is that “lengthening is a possible option for some skeletal dysplasias. If a family chooses to go down this path, they deserve to do so in the company of the best medical teams, specialized in providing comprehensive care before, during and after the process”. In this context, limb lengthening should be understood as a preference-sensitive intervention rather than a universal standard of care.

It is not the aim of this study to favor one strategy over the other. However, the data obtained from the model demonstrate that limb lengthening surgery in patients with achondroplasia is cost-effective at the WTP threshold considered [47] and, therefore, the procedure should be made available to any person with achondroplasia willing to undergo the operation.

As regards our selection of the Markov model, it was motivated by the fact that it is ideally suited to represent irreversible or long-standing conditions where patients transition between various mutually exclusive states, which is exactly the situation patients with achondroplasia are in [37]. Although a discrete-element simulation model might have been a valid alternative [61], it would most likely have required a much larger, difficult to compile, dataset. The states under the model, as well as the transition probabilities and their associated costs and utilities, were taken either from the literature [4, 40, 42–45] or from the clinical practice of the only unit specializing in achondroplasia in the Spanish Health System (AD-VVUH), apart from being discussed with representatives from the largest patient association in the country (Alpe Foundation).

Within this modeling framework, severe complications were represented as a separate health state, as they typically involve reoperation and follow a distinct clinical pathway. We considered that such events are more appropriately captured through an explicit state rather than as modifiers of another state.

As far as the comparators used are concerned, it could be argued that a thorough evaluation of any healthcare technology requires an individual analysis of all the options available. However, the pharmacological treatment available for the disease could not be included in the simulation. There are currently several studies underway aimed at analyzing the effects of various FGFR3-targeted therapies. Among them, those on vosoritide are the ones at a more advanced stage. Vosoritide is a recombinant c-type natriuretic peptide (CNP) analog designed with increased stability and a longer half-life. In patients with achondroplasia, it binds to the NPR-B receptor, antagonizing hyperactive FGFR3 signaling and promoting endochondral bone growth by stimulating chondrocyte proliferation and differentiation [27]. Although treatment with vosoritide offers promising results [27–29] it cannot, at least for the time being, be included as a comparator for methodological reasons. Firstly, because, until now, it has only succeeded in achieving height increases below 6 cm [29], which is far from allowing patients to reach the height necessary to obtain significantly better functional and quality-of-life outcomes [22, 31]. Secondly, because the only study analyzing the quality of life of patients treated with the drug [28] employed a specific scale for children of short stature (Quality of Life of Short Stature Youth) rather than a generic scale, as would have been desirable for an economic analysis [62–64]. To the best of our knowledge there is no way of automatically mapping the data from a specific to a generic scale. Finally, vosoritide was approved too recently to be sure about the costs involved in its use in a clinical environment [65], and its final market price is still uncertain as it is currently considered an orphan drug [25, 26]. It is to be expected that this or other similar comparators will be incorporated to economic evaluation studies in the near future. In fact, analyses have been carried out to evaluate the possibility of combining surgical and pharmacological treatment [66].

Also with respect to the comparators used here, it should be mentioned that although there are various limb lengthening protocols [31], the one analyzed in this study comprises two distinct stages [14]. The first one involves a simultaneous lengthening of both femora and tibias. The second (optional) phase consists in lengthening the upper limbs. Other protocols could lead to different conclusions. In fact, the protocol currently used by the AD-VVUH includes the prophylactic use of elastic intramedullary nailing following the lengthening procedure, which reduces the incidence of postoperative complications. This effect was not factored into the model due to the lack of up-to-date data.

The choice of a WTP threshold is also a matter for debate. Although an official threshold value has never been defined for Spain, the arbitrary figure of €30,000/QALY has often been used in the literature [67]. The most notable official effort to estimate a cost-effectiveness threshold in Spain was conducted by the Spanish Network of Health Technology Assessment Agencies, following a commission from the Ministry of Health. This work resulted in both an official report and a peer-reviewed publication, which suggested a threshold in the range of €20,000–€25,000 per QALY [68, 69]. However, these estimates were based on data from a period coinciding with the 2008 economic recession and were explicitly presented as subject to revision. More recent work has revisited these estimates, proposing a flexible double-threshold framework with a lower bound around €25,000/QALY and an upper bound around €60,000/QALY, within which additional contextual factors should be considered in decision-making [47]. In any case, given the ICER obtained in our study, none of these thresholds —not even the most restrictive— would change the decision regarding the cost-effectiveness of the intervention. Moreover, several factors in our case may support consideration of the upper threshold. Achondroplasia is a rare pediatric condition, the benefits of treatment are expected to be long-lasting, and the number of eligible patients is very small.

The cost-effectiveness plane scatter plot of the PSA showed a significant dispersion, which reflects the high variability across patient-reported health outcomes. This dispersion also indicates that, even at high WTP thresholds, the operative option may not be cost-effective in some of the iterations. An alternative way of representing this phenomenon would be by means of the cost-effectiveness acceptability curve (CEAC). In our case, given that limb lengthening never reaches a 100% probability of being the alternative with the highest net monetary benefit, the CEAC does not tend asymptotically to the unit value. However, the mean ICER of our simulations indicates that surgery is a cost-effective option at the selected WTP threshold, and the acceptability frontier confirms that the mean net monetary benefit is greater for the operative alternative when the WTP threshold is above €7,814/QALY, and that the mean net expected benefit is higher for the selected WTP threshold, confirming the cost-effectiveness of limb lengthening at that threshold. The uncertainty is also reflected by the EVPI curve, which does not go down abruptly below the WTP threshold equivalent to the mean ICER, suggesting the need to carry out further studies to obtain additional information on comparative costs and results. At any event, these findings should be interpreted in the light of the size of the target population and of the expected cost of the studies to be performed, with a view to determining whether the investment in additional studies is warranted.

In fact, as mentioned above, the budgetary impact of including limb lengthening in the Spanish Health System’s roster entails an increase in expenditure of only 8 million euros over 15 years. Moreover, it must be considered that this amount was calculated taking into consideration the worst-case expenditure scenario, where all patients with achondroplasia in the country choose to undergo the treatment. Clearly, a highly unlikely scenario. Indeed, when assuming that only 50% of the eligible population undergoes the procedure, the estimated budget impact is reduced to approximately 4 million euros over the same period, further supporting the affordability of the intervention.

To conclude, the authors would like to consider the strengths and weaknesses of this study as a way of facilitating a more critical interpretation of the findings presented.

Several limitations should be acknowledged. The study from which the utility estimates were derived has a relatively small sample size [42], and this may affect the generalizability of our results. It is also true that our model assumes that the quality-of-life improvement resulting from the surgical option persists throughout the subjects’ lifetime, which could lead to an overestimation of the total benefit. As regards costs, although the prices of the various resources are fixed, inaccuracies may have occurred when calculating the number of resources employed for each treatment alternative as a result of the retrospective nature of the cost analysis. Lastly, as there are no data in the literature for some of the parameters used in the study, the information was obtained from internal data from the AD-VVUH, introducing potential inaccuracies. To address this problem, a fully-fledged probabilistic analysis was carried out to reduce the potential effect of any estimation errors.

In terms of strengths, it must be said that limb lengthening is an uncommon treatment and that the study from which the utility values were obtained is, to our knowledge, the only one evaluating patients with achondroplasia in three different scenarios: no lengthening, lower limb lengthening, and all-limb lengthening. In addition, ours is, to our knowledge, the first study to analyze the cost-effectiveness of limb lengthening surgery, providing a basis for decision-making and a solid groundwork for future analysis.

## Conclusion

Limb lengthening surgery in patients with achondroplasia can be considered a cost-effective intervention according to the most commonly accepted willingness-to-pay thresholds in Spain and other Western countries.

## Data Availability

The datasets generated and/or analyzed for the current study are not publicly available due to their medical nature but may be obtained from the corresponding author on reasonable request.

## Abbreviations

AD-VVUH: Achondroplasia Department of the Virgen de la Victoria University Hospital
ALPE: Fundación Alpe Acondroplasia
CEAC: Cost-Effectiveness Acceptability Curve
CEAF: Cost-Effectiveness Acceptability Frontier
EQ-5D-Y: EuroQol 5 Dimensions – Youth version
EVPI: Expected Value of Perfect Information
FGFR3: Fibroblast Growth Factor Receptor 3
ICER: Incremental Cost-Effectiveness Ratio
INE: Instituto Nacional de Estadística
NICE: National Institute for Health and Care Excellence
PSA: Probabilistic Sensitivity Analysis
QALY / QALYs: Quality-Adjusted Life Year(s)
SAS: Servicio Andaluz de Salud
SNS: Sistema Nacional de Salud
